# Two cases of male patients followed for a classical form of congenital adrenal hyperplasia (CAH), presenting an azoospermia: analysis and review of the literature

**DOI:** 10.1186/s12610-019-0084-8

**Published:** 2019-03-12

**Authors:** Clélia Fouques, Imène Fatfouta, Sylvie Hieronimus, Jean-Louis Sadoul, André Bongain

**Affiliations:** 1 Department of Gynecology-Obstetrics, Archet 2 Hospital, 151 route Sainte-Antoine de Ginestière CS 23079, 06202 Nice, Cedex 3 France; 2 Departments of Endocrinology, Archet 2 Hospital, 151 route Sainte-Antoine de Ginestière CS 23079, 06202 Nice, Cedex 3 France

**Keywords:** Congenital adrenal hyperplasia, 21-hydroxylase deficiency- azoospermia - male infertility, Testicular adrenal inclusions, “TART” lesions, Hyperplasie congénitale des surrénales – Bloc en, 21-hydroxylase - Azoospermie - Infertilité masculine, Inclusions surrénaliennes testiculaires, Lésions “TART”.

## Abstract

**Background:**

Congenital hyperplasia of the adrenal glands is a rare pathology, which can have an impact on male fertility. We report 2 cases of azoospermia in patients followed for a classical form of congenital adrenal hyperplasia.

**Cases presentation:**

1st case: After 18 months of infertility of the couple, explorations showed a high level of ACTH on the hormonal biological analysis. A therapeutic strategy combining hydrocortisone with dexamethasone induced a normal semen analysis, and the female partner of the patient subsequently had three spontaneous pregnancies.

2nd case: After two years of infertility of the couple, explorations showed adrenal testicular inclusions invading the 4/5th of the testis with a hypergonadotropic hypogonadism, the therapeutic reinforcement did not allow the improvement of semen analysis.

**Discussion:**

Sertolian deficiency can be explained by: gonadotropic deficiency by excess of adrenal androgens and adrenal testicular lesions (risk of major spermatic alteration).

**Conclusion:**

Congenital hyperplasia of the adrenal glands is a rare pathology in the context of male infertility. A semen analysis could be performed after puberty and a semen preservation may be proposed.

## Abstrait

**Contexte:**

L’hyperplasie congénitale des surrénales est. une pathologie rare, qui peut avoir un impact sur la fertilité masculine. Nous rapportons 2 cas de forme classique d’hyperplasie congénitale des surrénales présentant une azoospermie.

**Présentation des cas:**

Premier cas: Après une infertilité de 18 mois du couple, les explorations biologiques montraient un taux élevé d’ACTH. Une modification du traitement combinant l’hydrocortisone à la dexaméthasone a permis une normalisation du spermogramme: trois grossesses spontanées ont été obtenues.

Second cas: Après une infertilité de 2 ans, les explorations révélaient des inclusions testiculaires surrénaliennes envahissant les 4/5 Emes des testicules avec un bilan retrouvant un hypogonadisme-hypergonadotrope et le renforcement thérapeutique n’a pas permis l’amélioration du spermogramme.

**Discussion:**

L’insuffisance Sertolienne peut être expliquée par: le déficit gonadotrope secondaire à l’excès d’androgènes surrénaliens et la présence de lésions testiculaires surrénaliennes (risque d’altération spermatique majeure).

**Conclusion:**

L’hyperplasie congénitale des glandes surrénales est. une pathologie rare dans le cadre de l’infertilité masculine. Un spermogramme pourrait être réalisé dès la puberté et une auto-conservation du sperme pourrait être proposée.

## Background

Congenital hyperplasia of the adrenal glands (CAH) causes infertility for both sexes. Spontaneous fertility of men with CAH is lower than that of the general male population [1, 2].

The most common etiology (at least 95%) was the enzyme deficiency in 21-hydroxylase [3, 4], due to a CYP21A2 gene mutation. The 21-hydroxylase deficiency is responsible for a cortisol and aldosterone deficiency, and an increase of adrenal steroids upstream of the block, causing an androgens accumulation [1].

This pathology is very rare in newborns, it can be revealed at that age in its classical form with an incidence of 1/10000 to 1/15000 [5].

The most frequent forms (Incidence 1/1000) are diagnosed in adults, they are thermed as Non Classical 21-hydroxylase deficiency and enzymatic alteration is much more moderate [6].

We report the cases of Mr. B and Mr. D followed since childhood for a classical form of deficiency in 21 hydroxylase, with primary infertility in a context of azoospermia.

## Cases presentation

### First case

Mr. B and Mrs. B, respectively 28 and 26 years old, attended the Department of Endocrinology of the Hospital of Nice for an 18-month primary infertility.

Mrs. B, who had never been pregnant, with no significant medical past history, reported a oligomenorrhea, and presented clinical and biological signs suggesting hyperandrogenism: the diagnosis of polycystic ovarian syndrome was made.

Mr. B had a history of classical salt wasting form of CAH diagnosed since the first days of life. He did not smoke.

Sequencing of the entire CYP21 gene found a homozygous profile for a missense mutation, p. Asn387Ile (c. 1160a > T) in Exon 9. No CYP21 mutations were found in Mrs. B.

Mr. B received a daily dose of 25 mg hydrocortisone as supplementation treatment (15 mg in the morning, 10 mg in the evening) and 75 μg fludrocortisone (50 μg in the morning, 25 μg in the evening). The adrenal CT-scan did not show any adrenal abnormality.

Regarding the infertility assessment, azoospermia was confirmed twice by using a centrifugation pellet (Table 1).

**Table 1:** Case 1: Semen analysis before and after treatment adaptation

Semen analysis	Before treatment adaptation	After treatment Adaptation
Volume (mL)	3.2	4.3
pH	7.9	7.9
Numeration (Million/mL)	0	19
Sperm progressive motility (a + b) (%)	0	34
Sperm vitality (%)	0	5
Sperm preparation (Million motile sperm/mL)	Not done	1.8

Sperm progressive motility (a + b): a: sperm rapidly progressive, b: sperm slowly progressive

The hormonal analysis showed a poor control of the adrenocorticotropin (ACTH) secretion (increased level at 1122 ng/L [normal range: 0–52 ng/L]), total testosterone levels were normal at 5.7 μg/L [normal range: 2.5–10.0 μg/L], ∆ 4-Androstenedione was increased at 15.6 μg/L [normal range: 0.5–2.6 μg/L] with collapsed gonadotropin levels. The remainder of the analyses showed a major increased in 17-hydroxyprogesterone level (17-OHP) at 72 μg/L [normal range: 0.3–2.2 μg/L] (Table 2).

**Table 2:** Case 1: Hormonal parameters before and after treatment adaptation

Hormonal parameters	Before treatment adaptation	After treatment Adaptation
ACTH (ng/L)	1122	8
Total testosterone (ug/L)	5.7	3.2
∆4-Androstenedione (ug/L)	15.6	0.3
FSH (IU/L)	< 0.5	7.5
LH (IU/L)	< 0.5	2.5
17-OHP (ug/L)	72	0.9

The genital clinical examination was strictly normal, however, testicular ultrasound revealed multiple lesions suggestive of less than one-centimeter adrenal intra-testicular residues, the testicular volume was considered to be normal.

A treatment was initiated with 25 mg of hydrocortisone in the morning and 0.5 mg of dexamethasone at bedtime.

Over 4 months, ACTH and 17-OHP levels significantly decreased (Table 2), pituitary secretions of the gonadal axis and the testicular exocrine function went back to normal levels, inducing a normal semen analysis (Table 1), according to 2010 WHO Guidelines [7].

After 6 months of treatment, Mrs. B became pregnant, the pregnancy was complicated by an intrauterine growth retardation at 37 weeks of amenorrhea. She gave birth to a little girl of 2000 g and measuring 43 cm and now the little girl is doing well with a good weight evolution.

An annual control of the biological levels and semen analysis showed a complete stabilization for these parameters. Testicular ultrasound did not show any evolution of adrenal residues. Then a semen preservation was carried out.

After one year, due to significant weight gain (20 kg/ BMI 37.9 kg/m^2^) dexamethasone was stopped. Two years later, the couple wanted a second child, so drug treatment by dexamethasone and hydrocortisone was initiated. Two years after treatment modification, it resulted in a new pregnancy ending by a spontaneous miscarriage. Two months after the miscarriage, the female partner of the patient was pregnant again and she is currently at 24 weeks of amenorrhea of pregnancy.

### Second case

Mr. D and Mrs. D 33 and 32 years old respectively attended the Reproductive Medicine Department at the Hospital of Nice for a 2-year primary couple infertility.

Mrs. D had no particular medical history, and her hormonal biological levels and the ultrasound examination showed a good ovarian reserve.

Mr. D had been followed since birth for a classical salt-wasting form of CAH. He carries a compound heterozygous mutation (IVS2–13 A/C > G in the intron 2 and for the mutation Q318X), with a normal karyotype 46,XY. Mrs. D did not present any mutation.

Mr. D had been traited by hydrocortisone and fludrocortisone since birth; this treatment was replaced by triptoreline from 9 to 14 years of age. The fludrocortisone was stopped at the age of 30. He was treated with hydrocortisone (30 mg in the morning-20 mg at noon-10 mg in the evening). He had no other significant medical history and did not smoke.

Regarding the fertility assessment: the semen analysis showed a confirmed azoospermia twice by using a centrifugation pellet (Table 3).

**Table 3:** Case 2: Semen analysis before and after treatment adaptation

Semen analysis	Before treatment adaptation	After treatment Adaptation
Volume (mL)	4	3.5
pH	7.9	7.9
Numeration (Million/mL)	0	0
Sperm progressive motility (a + b) (%)	0	0
Sperm vitality (%)	0	0
Sperm preparation (Million motile sperm/mL)	Not done	Not done

Sperm progressive motility (a + b): a: sperm rapidly progressive, b: sperm slowly progressive

FSH and LH were increased (respectively: 16.9 IU/L and 9.8 IU/L [normal range: FSH 1.5–18 IU/L, LH 1.5–9 IU/L]) suggesting a secretory-type azoospermia. Testosterone was normal (4.1 μg/L) compared with high gonadotropin (FSH 16.6 IU/L, LH 6.4 IU/L) with a major elevation of Δ4-androstenedione (30.7 μg/L), 17-OHP (60 μg/L) (Table 4).

**Table 4:** Case 2: Hormonal parameters before and after treatment adaptation

Hormonal parameters	Before treatment adaptation	After treatment Adaptation
ACTH (ng/mL)	Not done	Not done
Total testosterone (ug/L)	4.1	1.9
∆4-Androstenedione (ug/L)	30.7	0.7
FSH (IU/L)	16.6	21.2
LH (IU/L)	6.4	12.5
17-OHP (ug/L)	60	0.6

Treatment was then modified: hydrocortisone 20 mg in the morning, and 10 mg at noon and dexamethasone was initiated with a daily dose of 0.5 mg, one tablet in the evening.

**Fig. 1:**
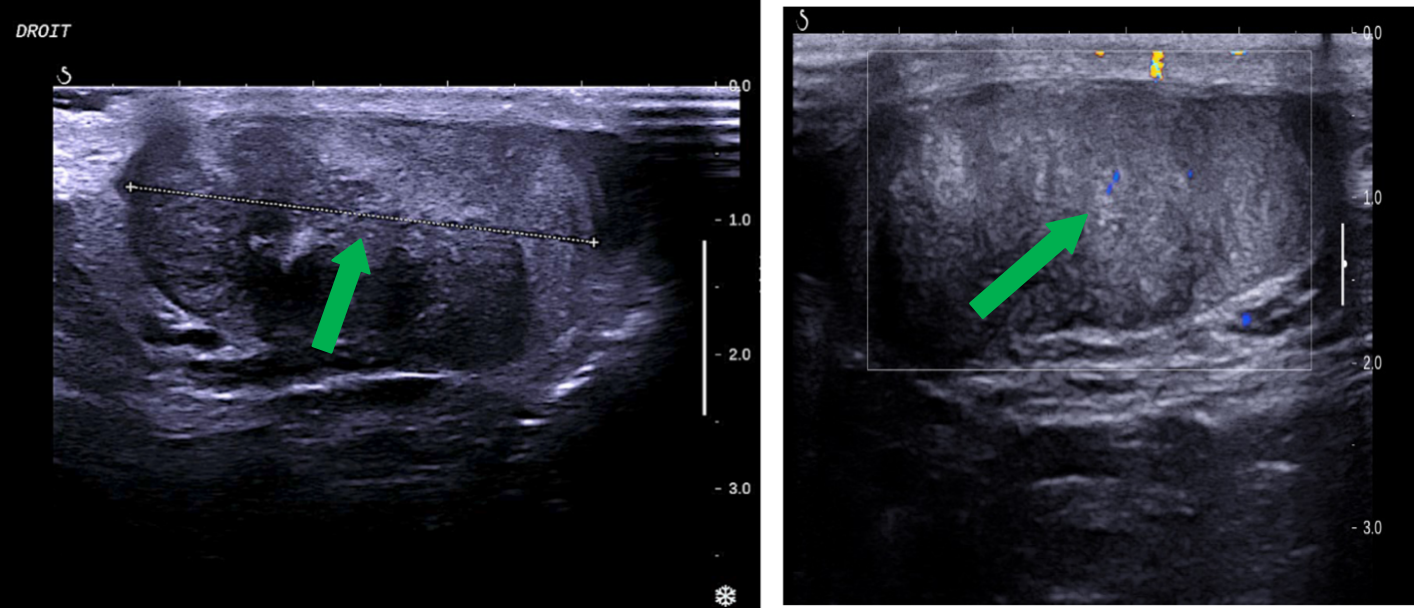
Scrotal ultrasounds of the right testis from second case. Legend. Arrows indicate Testicular Adrenal Rest Tumors (TART)

**Fig. 2:**
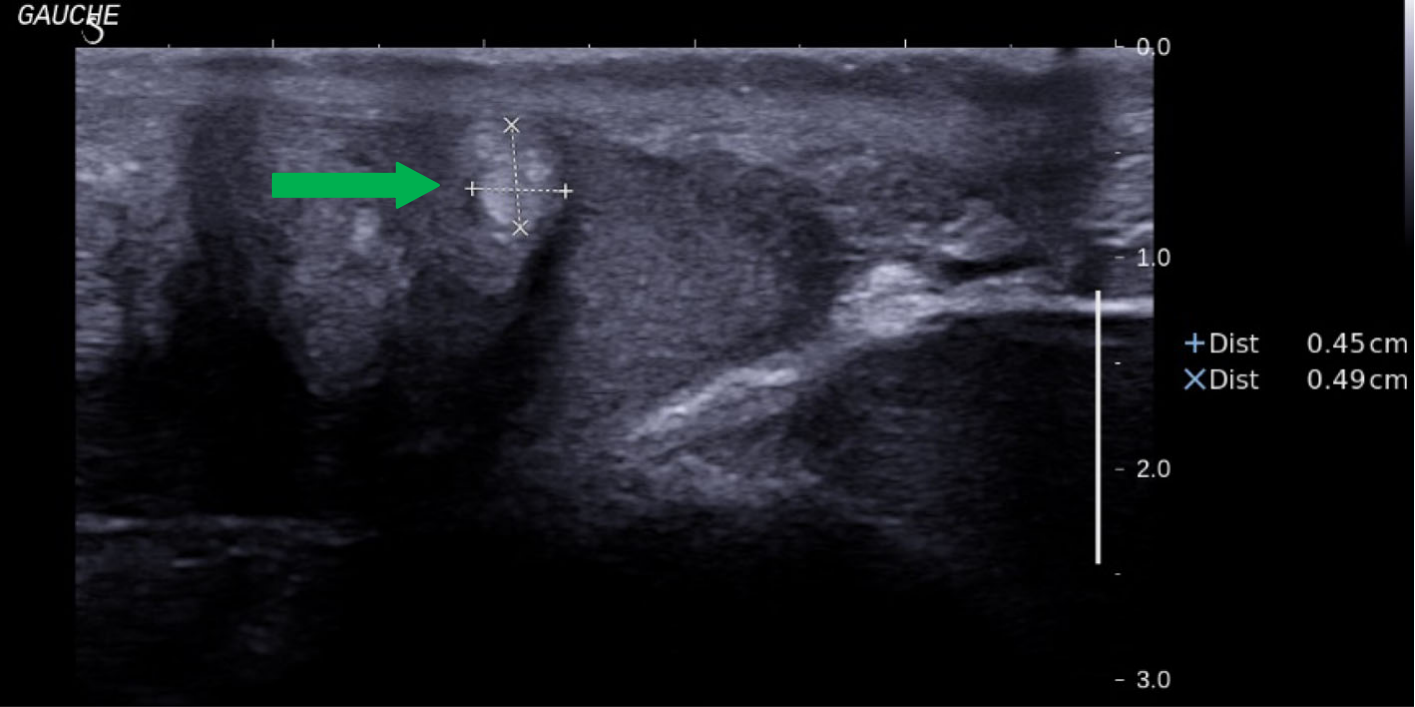
Scrotal ultrasounds of the left testis from second case. Legend. Arrow indicates Testicular Adrenal Rest Tumors (TART)

Clinically, he had stony testes and testicular ultrasound examination showed an heterogeneous echostructure of the two glands with only one band of normal testicular parenchyma, stage V (Figs. 1 and 2).

**Fig. 3:**
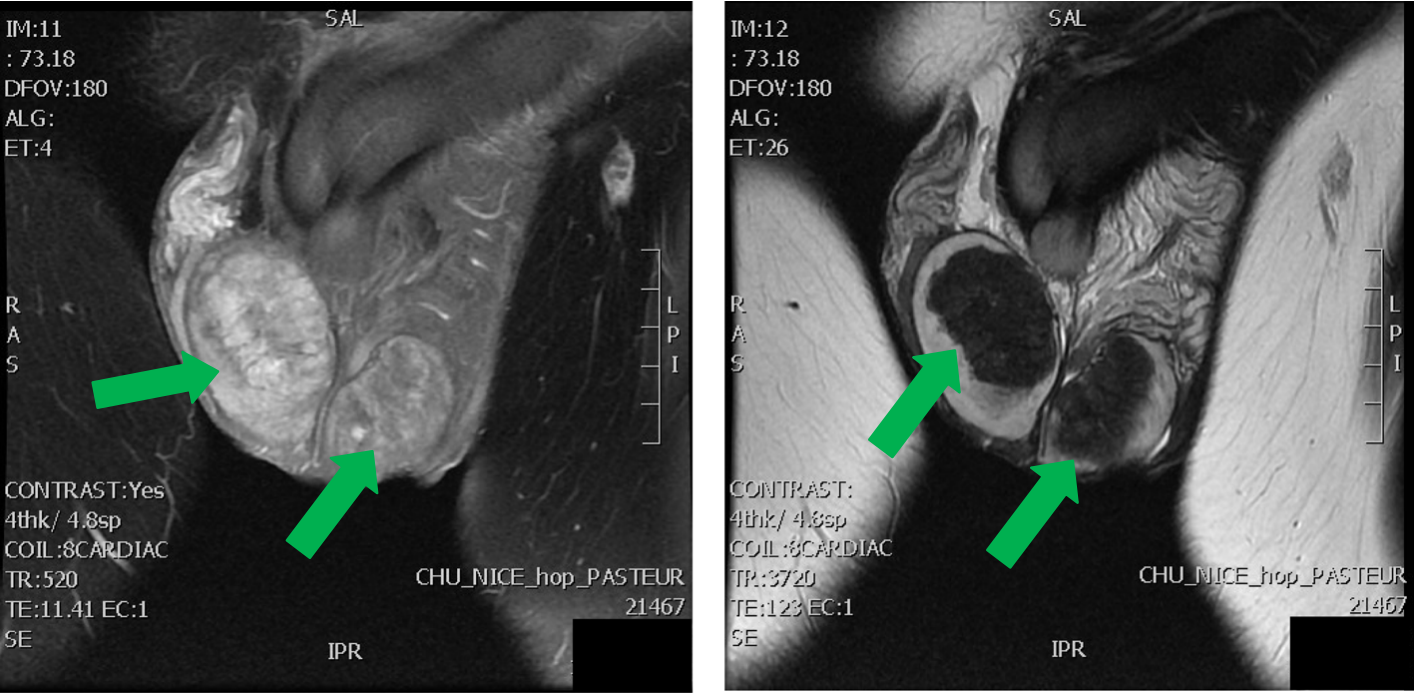
Scrotal MRI of the testis from second case Legend. Arrows indicate Testicular Adrenal Rest Tumors (TART)

A testicular MRI was carried as slight volumetric evolution of a right adrenal inclusion was showed during the ultrasound examination follow-up. MRI confirmed the typical appearance of large intra-testicular adrenal inclusions (Fig. 3). The adrenal CT-scan did not show any adrenal abnormality.

Treatment changes allowed a total control of 17-OHP (0.6 μg/L) and Δ4-androstenedione (0.7 μg/L) unmasking a hypergonadotropic-hypogonadism: total testosterone 1.9 μg/L, increased levels of FSH and LH up to 21.2 IU/L and 12.5 IU/L respectively (Table 4).

Three semen preservation were proposed but no spermatozoa were found.

After one year of treatment, a bilateral exploration with testicular biopsy was proposed to identify focal spermatogenesis focus, and no sperm cell could be found.

The sperm donation was purposed to the couple, but they refused; as they had split up.

## Discussion

The 21-hydroxylase deficiency is responsible for 95% of the CAH, it is a disease with an autosomal recessive transmission and there are more than 200 mutations known [8].

Deficiency in 21-hydroxylase results in a cortisol deficiency, an increase in ACTH secretion upstream of the block. This results in an increase of cortisol precursors, including 17 – OHP and a deviation towards the pathway of adrenal androgens, including the testosterone-metabolized ∆ 4-androstenedione: producing excess androgens.

If necessary an increase of 17OHP beyond the 10 ng/mL threshold after stimulation with ACTH makes the diagnosis, which leads to consider a molecular study of CYP21A2 gene and a family survey.

### Male fertility

The paternity rate in patients with a 21-hydroxylase deficiency is lower (51%) than in the general population (79%). However, when a pregnancy is obtained, it is spontaneous in 89% of cases; 11% of pregnancies are obtained by In Vitro Fertilization (IVF) [1]. The paternity rate is lower in the classical form with salt wasting [2].

Semen parameters were in the normal range (according to the WHO Guidelines 2010) in 34% of the cases. In 24% of cases, a moderate oligozoospermia (sperm count: 5–15 millions/mL) was found, and in 42% of cases, severe oligozoospermia (less than 5 millions/mL) or azoospermia [1].

Two mechanisms may explain the Sertolian deficiency:

- Gonadotropic deficiency related to excess androgens adrenal

Adrenal androgens excess could be responsible for a negative feedback on gonadotropin secretion. The increase in progesterone upstream of the block could also induce a negative feedback on the hypothalamic-pituitary axis. Gonadotropic deficiency results in alteration of spermatogenesis [1, 10, 11]. The total testosterone level is usually normal, sometimes lowered [1].

b. Testicular inclusions: TART lesions (Testis Adrenal Rest Tumor) [12–16]

The rate of spontaneous pregnancy in men with intra-testicular lesions is lower [1]: intra-testicular lesions are a risk factor for major spermatic alteration (severe oligozoospermia or even azoospermia) [1, 18].

The testicular function is altered, especially in the case of larges inclusions, up to the excretory pathways compression or to the hypergonadotropic hypogonadism. The testis tissue destruction resulted in a significant increase in FSH and a decrease in inhibin B [1]. Patients with testicular stunting have more severe sperm abnormalities [1].

The search for testicular lesions by testicular ultrasound should be done before puberty and then regularly in adulthood, even if the clinical examination is normal. Testicular ultrasound is the reference exam and the ultrasound characteristics of CAH are: multifocal, bilateral, confluent, attenuating, non-calcification inclusions.

The first-line substitution treatment in the 21-hydroxylase deficiency is hydrocortisone in monotherapy. Although the studies are controversial on the therapeutic equilibrium and prevalence of intra-testicular lesions [9, 16], these lesions may regress with glucocorticoid treatment wich leads to improving semen parameters [12, 17]. When intra-testicular lesions are present the addition of dexamethasone treatment may be discussed to improve the semen parameters [12].

### Discussion of the two clinical cases

Two phenomena explain the initial azoospermia of our patients.

Firstly, the gonadotropic deficiency causing the absence of spermatogenesis [1, 9, 10].

In the first case: the enhancement of the substitution treatment very quickly normalized gonadotropin and peripheral steroid levels with the main consequence: a resumption of spermatogenesis and 3 spontaneous pregnancies.

In the second case, drug treatment allowed a total control of 17-OHP and ∆ 4-androstenedione, but this control has highlighted a hypergonadotropic hypogonadism induced by the destruction of testicular parenchyma by adrenal inclusions.

Secondly, the presence of testicular inclusions of adrenal tissue [1, 6]. Indeed a prevalence of such inclusions was reproted to range from 40 to 95% of the cases and their impact on fertility was confirmed [1, 11, 18].

The current treatment remains prevention by early detection, when the lesions remain susceptible to the therapeutic increase. The issue of fertility preservation seemed to be a careful option [1, 9, 18].

Concerning the first patient, the inclusions were not altered by the treatment changes but did not at this stage play a negative role for the resumption of the spermatogenesis. A semen preservation was carried out.

For the second patient, the magnitude of the lesions resulted in an almost total destruction of the testicular parenchyma inducing a secretory azoospermia and a hypergonadotropic hypogonadism and no sperm was found by testicular exploration.

## Conclusion

CAH with a 21-hydroxylase deficiency is a rare pathology but a documented etiology for male infertility. Presented in its classical form, the link between spermatogenesis alteration and poor therapeutic control can be summarized in a simple way.

When intra-testicular lesions are advanced, consecutive destruction of Leydig cells may result in hypergonadotropic-hypogonadism. The use of a sperm donation is then a therapeutic alternative that should be proposed to couples.

Regular follow up by a reference center is recommended.

In view of the frequency of these lesions, and the absence of systematic clinical signs, an ultrasound examination in search of testicular lesions must be done at the end of puberty and then regularly in adulthood.

Any deficiency must be taken into account and treated by a control of long term therapeutic compliance and a reinforcement if necessary of the associated adrenal androgens suppression therapy.

A semen analysis could be proposed after puberty to detect a spermatic alteration, mostly if TART lesions are detected. Since 2011, the French National Authority for Health (HAS) recommends a semen preservation in all patients with a classical form due to the high rate of oligozoospermia, or even azoospermia.

### Normal ranges of the laboratory

ACTH: 0–52 ng/L.

FSH: 1.5–18 IU/L.

LH: 1.5–9 IU/L.

∆4-Androstenedione: 0.5–2.6 μg/L.

Testosterone: 2.5–10.0 μg/L.

17-OHP: 0.3–2.2 μg/L.

## Abbreviations

17-OHP: 17-hydroxyprogesterone
ACTH: Adrenocorticotropin
BMI: Body Mass Index
CAH: Congenital Adrenal Hyperplasia
CYP21: Cytochrome P21
FSH: Follicle Stimulating Hormone
HAS: French National Authority for Health
IVF: In Vitro Fertilization
LH: Luteinizing Hormone
TART: Testis Adrenal Rest Tumor
